# Chitosan as an Outstanding Polysaccharide Improving Health-Commodities of Humans and Environmental Protection

**DOI:** 10.3390/polym15030526

**Published:** 2023-01-19

**Authors:** Lorenzo A. Picos-Corrales, Ana M. Morales-Burgos, Jose P. Ruelas-Leyva, Grégorio Crini, Evangelina García-Armenta, Sergio A. Jimenez-Lam, Lidia E. Ayón-Reyna, Fernando Rocha-Alonzo, Loranda Calderón-Zamora, Ulises Osuna-Martínez, Abraham Calderón-Castro, Gonzalo De-Paz-Arroyo, Levy N. Inzunza-Camacho

**Affiliations:** 1 Facultad de Ingeniería Culiacán, Universidad Autónoma de Sinaloa, Ciudad Universitaria, Culiacán 80013, Sinaloa, Mexico; 2 Facultad de Ciencias Químico Biológicas, Universidad Autónoma de Sinaloa, Ciudad Universitaria, Culiacán 80013, Sinaloa, Mexico; 3 Laboratoire Chrono-Environnement, UMR 6249, UFR Sciences et Techniques, Université de Franche-Comté, 16 Route de Gray, 25000 Besançon, France; 4 Departamento de Ciencias Químico Biológicas, Universidad de Sonora, Hermosillo 83000, Sonora, Mexico; 5 Facultad de Biología, Universidad Autónoma de Sinaloa, Ciudad Universitaria, Culiacán 80013, Sinaloa, Mexico; 6 Unidad Académica Preparatoria Hermanos Flores Magón, Universidad Autónoma de Sinaloa, Culiacán 80000, Sinaloa, Mexico

**Keywords:** chitosan, antimicrobial agent, nutraceutical formulations, anticancer drug formulations, edible coatings, elicitors, textiles, food packaging, catalytic scaffolds, bioflocculation

## Abstract

Public health, production and preservation of food, development of environmentally friendly (cosmeto-)textiles and plastics, synthesis processes using green technology, and improvement of water quality, among other domains, can be controlled with the help of chitosan. It has been demonstrated that this biopolymer exhibits advantageous properties, such as biocompatibility, biodegradability, antimicrobial effect, mucoadhesive properties, film-forming capacity, elicitor of plant defenses, coagulant-flocculant ability, synergistic effect and adjuvant along with other substances and materials. In part, its versatility is attributed to the presence of ionizable and reactive primary amino groups that provide strong chemical interactions with small inorganic and organic substances, macromolecules, ions, and cell membranes/walls. Hence, chitosan has been used either to create new materials or to modify the properties of conventional materials applied on an industrial scale. Considering the relevance of strategic topics around the world, this review integrates recent studies and key background information constructed by different researchers designing chitosan-based materials with potential applications in the aforementioned concerns.

## 1. Introduction

**Figure 1:**
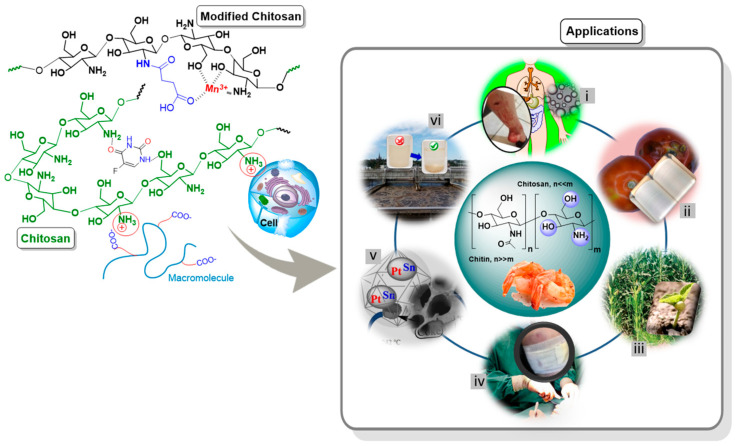
Scheme related to the interaction of the chitosan backbone with small molecules, macromolecules, cell, and inorganic substances, as well as the different applications such as issues in medicine (i), postharvest conservation and food packaging (ii), agriculture (iii), (cosmeto-)textile industry (iv), catalysis (v), and water treatment (vi).

Chitosan is an outstanding biodegradable and biocompatible polysaccharide, recognized as safe and produced from an abundant and renewable source (chitin). It is highly effective, which has been well demonstrated for various applications including food, nutraceuticals, pharmaceuticals, medicine, agriculture, textiles, pulp and paper, biotechnology, cosmetics and environmental chemistry, as previously documented by Morin-Crini et al. [1]. There are indeed more than 2000 applications of chitin and chitosan. Chitosan is a versatile and ideal material due to its intrinsic properties (e.g., non-toxicity, cationic character, and antibacterial activity) and particular chemical structure. This linear aminopolysaccharide provides reactive functional groups (-OH and -NH_2_ with a high percentage of nitrogen compared to substituted cellulose) that promote suitable chemical interactions with small molecules, ions, macromolecules, cell membranes/walls, and metal surfaces by electrostatic interactions, hydrogen bonding, hydrophobic interactions, and chelation (see Figure 1) [2,3,4,5,6,7]. Primary amino groups of chitosan play a key role in its different properties, such as its cationic nature in an aqueous acidic environment, controlled chemical interactions, mucoadhesion, in situ gelation, and antimicrobial activity [8]. In aqueous systems, the interactions between this aminopolysaccharide and target substances/living organisms depend on the pH of the medium and on the chitosan characteristics such as molecular weight (*M_W_*) and degree of deacetylation (DD). As demonstrated from zeta potential (ζ) measurements, chitosan is stable in solutions of pH < 6 (ζ > 15 mV), which is driven by the protonation of the amino groups (pKa ~ 6.4; depending on DD and *M_W_*); the isoelectric point (ζ = 0) of this macromolecule can be found within the pH range 6.9–7.7; and at higher pH, the polymer backbone becomes negatively charged. Thus, at pH between 6.5 and 8, the chitosan chains show ζ = 0 or near to this value, and therefore electrostatic interactions are limited [9,10,11]. However, the chitosan backbone can be modified to adjust its physicochemical properties (e.g., water solubility) and control its chemical interaction according to the application requirements [12,13,14], boosting the performance. Indeed, unlike chitin and cellulose, the presence of amino groups allows to perform chemical reactions specific to this reactive site. Depending on the desired goal, chitosan can be used either as the main component [15,16] or as an adjuvant along with other materials [17,18] for specific purposes. Another interesting property is its versatility, as chitosan can be used in different technological forms, such as solutions, powders, fibers, and films, depending on the intended application.

Regarding its interaction mechanisms, it has been stated that chitosan binds to small molecules like doxorubicin (DOX) or 5-fluorouracil (5FU), where electrostatic repulsion with positively charged DOX plays a key role and attractive interactions (by hydrogen bonds) are stronger with chitosan in a more deprotonated state [19], whereas hydrogen bonds are the principal interaction when non-ionized molecules (5FU) are involved [20]. Thus, chitosan-based therapeutic formulations can be designed to improve bioavailability and half-life, achieving a longer circulation time and sustained release [21], and reducing dosage requirements and adverse effects on non-target tissues. In the textile industry, the affinity of chitosan with dyes is highly relevant because a lower amount of dye is required and the dye release to the natural environment is reduced [22]. In agriculture, controlled release of pesticides can be reached with good bioactivity on the target plant, avoiding/reducing adverse impacts on the non-target plants and environmental contamination [23]. As an example of interaction with macromolecules, the major constituents of the intestinal mucus layer are the glycoproteins called mucins, and their interaction with chitosan can occur via electrostatic interactions, hydrogen bonding, and hydrophobic interactions [24]. In the human body, the amino groups of chitosan could bind to the sialic acid of mucins, triggering a better mucoadhesion [25]. In the case of interactions with cell membranes/walls, cationic chitosan perturbs the negative cell envelope of microorganisms. Thus, chitosan binds on a microbial cell wall preventing nutrients from entering the cell, alters the cell permeability, and could act as a metal chelator that inhibits microbial growth [5,26]. Therefore, this polymer is an antimicrobial agent that also stimulates the wound healing process, improves the antimicrobial and physical properties of textile fibers [27], provides an antimicrobial character and film-forming property for food packaging materials with adjusted tensile strength and elongation at break [28], and is a potential biostimulant and elicitor in agriculture [29]. For inorganic components involving metal surfaces, their interaction with the -NH_2_ and -OH groups of chitosan can be attributed to the strong chelating interaction between the lone-pair electrons of O or N atoms and empty d-orbits of the metal [30]; this is an advantage in processes such as the preparation of 3D-macroporous scaffolds with in situ formed metal nanoparticles (catalysis) [31] and the removal of heavy metal ions from water (water treatment) [6]. 

Chitosan is a material with outstanding physical, chemical, and biological properties that are valuable for a variety of uses. It is a polysaccharide that can help overcome drawbacks in medicine, postharvest conservation and food packaging, agriculture, the (cosmeto-)textile industry, catalysis, and water treatment (Figure 1), thus improving human health, protecting the environment, and sustaining a proper lifestyle. Hence, this review attempts to integrate the current efforts of different researchers working with chitosan in the aforementioned applications.

## 2. Antibacterial and Fungicide Power of Chitosan

**Figure 2:**
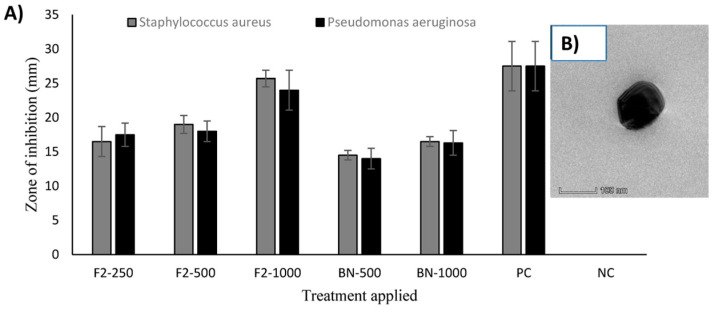
Antimicrobial activity of chitosan-curcumin nanoparticles against *Staphylococcus aureus* and *Pseudomonas aeruginosa* (**A**). The optimized formulation showed well-defined morphology (**B**). The figures were taken and combined from the literature [43].

It is well known that chitosan can inhibit the growth of bacteria (gram-positive and gram-negative) and fungi. However, this ability can vary according to many factors including the DD, the *M_W_*, and the molecular chain configuration, which affect the physicochemical properties of the macromolecules [32]. Moreover, differences in the target microorganism can contribute to this variability. Metabolomics analysis in *Listeria innocua* has provided evidence that the initial and the most important targets of chitosan are the cell membrane and cell wall. The cationic nature of this macromolecule might enable the interaction with these negatively charged organelles causing its disruption and ultimately contributing to the antibacterial effect of chitosan [33]. It has been reported that minimum inhibitory concentration values between different species of *Candida* spp. vary, probably due to negative charge density and composition of the cell wall [34]. Furthermore, studies suggested that chitosan uptake and its antifungal activity against *Penicillium expansum* (a fungal pathogen) are dependent on clathrin-mediated endocytosis [35]. To improve the antimicrobial power of chitosan, modifications of the backbone structure of this polysaccharide have been carried out. For instance, chitosan Schiff base derivatives, obtained via coupling chitosan with indole-3-carboxaldehyde, reached inhibition rates of 99% and 92% in gram-positive (*Staphylococcus aureus* and *Bacillus cereus*) and gram-negative (*Escherichia coli* and *Pseudomonas aeruginosa*) bacteria, respectively. The authors suggested that this was the result of an increased cationic capacity and better hydrophilicity of the polymer chain, as compared with non-modified chitosan [36]. On the other hand, in fungi, hydrophobicity is generally associated with better antifungal activity. Substitution reactions with diethylaminoethyl and dodecyl groups on chitosan generated macromolecules with amphiphilic properties, which increased their capacity to form hydrophobic interactions with the fungal cell wall and resulted in a better growth inhibition index of *Aspergillus flavus*, a human pathogen that can infect crops; moreover, a lower molecular weight led to higher inhibition [37]. Similarly, double Schiff bases bearing halogeno-benzenes were tested on *Botrytis cinerea* and showed increased antifungal effect (inhibitory indices > 95% at 1 mg mL^−1^) due to the strong electron-withdrawing property of halogens and the hydrophobicity, although imine groups might also have contributed to cell death, acting as chelators and affecting the uptake of essential metals [38]. Because microbial resistance became a major health problem worldwide, the efficacy of chitosan toward inhibition and re-sensitization of pathogens is being explored. In this regard, a biodegradable and biocompatible chitosan-derived cationic polymer (2,6-diamino chitosan) was verified as effective on bacteria of clinical importance and their multidrug-resistant strains. This notable outcome may be owed to the improved cationic power of chitosan by the incorporation of additional amino groups to its chemical structure, which triggered better adsorption onto the membrane of these bacteria [39]. Furthermore, disk diffusion assays have demonstrated that combination therapy of chitosan with antifungal drugs (e.g., fluconazole) exhibits remarkable synergistic inhibitory effects not only on sensible but also on resistant clinical strains of *Candida* species [26]. Another strategy that has been designed to tackle antimicrobial resistance is the use of chitosan nanoparticles for biomedical purposes, such as the growth inhibition of sensible and resistant *Nisseria gonorrhoeae*. In areas such as aquaculture, where better options for the treatment and prevention of infections are necessary, chitosan nanoparticles have yielded a wide range of antimicrobial activity in bacteria and fungi isolated from *Nile tilapia*, which is a fish of global importance [40,41]. A sponge-like material obtained from thymine-modified chitosan derivatives has enhanced the treatment of wounds and tissue regeneration, and when the degree of substitution increased from 0 to 0.62, the minimal inhibitory concentrations (MICs) decreased from 64 to 16 μg mL^−1^ for *Acinetobacter baumannii*. In this perfect example, the material can confer protection against nosocomial pathogens that commonly infect wounds [42]. Figure 2 represents an illustrative example of antimicrobial activities of chitosan-based formulations developed for skin tissue regeneration, using chitosan-curcumin nanoparticles and different treatments: 250 µg, 500 µg, 1000 µg, BN = Blank nanoparticles, PC = positive control (Gentamicin), NC = negative control (distilled water); from that, antimicrobial activity against *Staphylococcus aureus* and *Pseudomonas aeruginosa* was studied. As a result, F2-1000 (1000 µg nanoparticles) reached similar effects as compared to the positive control and showed higher activity than blank nanoparticles [43]. 

Thus, the effectiveness of chitosan as an antimicrobial agent has been well demonstrated and projects it as an advanced polysaccharide with unique properties toward the development of commercial pharmaceutical formulations.

## 3. Biomedical Application of Chitosan: Encapsulation of Active Molecules

### 3.1. Phytochemical Protection Using Chitosan: Nutraceutical Formulations

**Figure 3:**
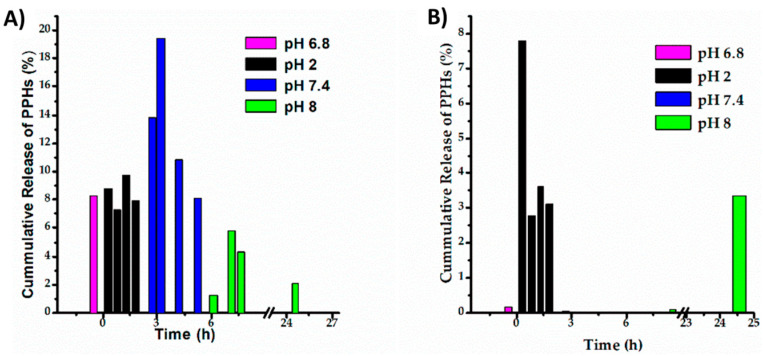
Cumulative release of phenolic compounds present in Oregano (*Lippia graveolens*) and encapsulated in chitosan-*b*-PPEGMA (**A**) and single chitosan (**B**) matrices. The trials were performed in simulated salivary fluids. The figure was taken from the literature [55].

Natural products such as plant-derived nutraceuticals, commonly used as functional ingredients in food, represent a suitable option to enhance treatments for cancer, diabetes, bacterial infections, and other diseases [44,45]. However, nutraceuticals exhibit limited gastrointestinal permeability and are susceptible to certain conditions such as degradation reactions and changes in pH and temperature [46]. These disadvantages affect the performance of the active agents during their oral administration. In this sense, colloidal carriers based on chitosan have been developed to protect these substances, obtaining micro- and nano-particles with biocompatible and biodegradable characteristics [47]. The interactions between the aminopolysaccharide and nutraceutical compounds can evolve from hydrogen bonds and electrostatic interactions. Specifically, it has been reported that the electrostatic interaction between curcumin and chitosan nanoparticles has a strong correlation with the number of intermolecular hydrogen bonds [48]. As it is well known, -NH_2_ groups onto this polysaccharide allow its chemical derivatization and grafting to improve its physicochemical properties [49], where one of the objectives is to enhance the water solubility of this macromolecule, increasing the permeability of hydrophobic nutraceuticals, rising the potential of loaded particles as carriers for oral delivery [50]. Regarding mucins, which are glycoproteins that are the major constituents of the intestinal mucus layer, their interaction with the chitosan backbone depends on the properties of both (mucins and the polymer) and can occur via electrostatic interactions, hydrogen bonding and hydrophobic interactions [24]; the amino groups of chitosan can bind to the sialic acid of mucins resulting in a better mucoadhesion [25], as well as the change in the surface’s charge (from negative to positive) of chitosan could trigger an increase in the adhesion of loaded particles on the intestinal mucosa [25,51]. In this topic, several studies have reported the development of nutraceutical formulations for oral administration using chitosan and modified chitosan. For instance, researchers reported that apocynin (an anti-inflammatory phytochemical extracted from roots of *Apocynum cannabinum*) can be encapsulated in a w/o/w emulsion with non-modified chitosan, producing microparticles with suitable sizes around 326 nm (PDI = 0.201) and an encapsulation efficiency of 45%, and providing controlled drug release under in vitro gastrointestinal conditions (9.7% and 28.8% at a pH of 1.2 and 6.8, respectively). Furthermore, good stability under storage and large periods of absorption by the oral route were registered [52]. Conversely, the same guest molecule (apocynin) was loaded in a platform from modified chitosan oligosaccharide crosslinked with tripolyphosphate (TPP), resulting in a higher particle size (436 nm; PDI = 0.39), lower encapsulation efficiency (35%), and higher drug release percentage at pH 1.2 (44%) in comparison to the previous report using non-modified chitosan. Moreover, the authors indicated that this carrier system presented good stability and enhanced efficacy in longer-time oral administration for gastric ulcers [53]; namely, the modification of chitosan can be useful for specific purposes. Similarly, nanogels from chitosan grafted by *ρ*-coumaric acid loaded with *Syzygium aromaticum* essential oil (particle size = 255 nm) exhibited a slow in vitro release at pH 7.4 (87.5% after 16 days); in addition, the system resulted in increased antioxidant activity and great potentials for boosting the antibacterial activity of the native essential oil [54]. Another example is the preparation of chitosan-polycaprolactone nanoparticles loaded with thymoquinone (particle size = 182 nm), which showed a suitable control release of up to 24 h in simulated intestinal fluids, providing excellent mucoadhesion properties and improved oral bioavailability [25]. In other work, when compared to chitosan, copolymers based on chitosan and polyethylene glycol methyl methacrylate (PEGMA) loaded with phenolic compounds (extracted from oregano) resulted in lower particle size (458 nm), more controlled release patterns in response to pH changes, and higher protection for the active agents in simulated gastric conditions (see the cumulative release profiles in Figure 3). However, this study revealed that the carrier system prepared with non-modified chitosan (particle size = 1106 nm) had better loading efficiency and was more stable (zeta potential = 50 mV) than the block copolymers-based system (zeta potential = −15 mV) [55]. In the same way, the encapsulation of quercetin in platforms developed from chitosan and soybean polysaccharides has resulted in nanoparticles with appropriate size (close to 24 nm) for targeting specific cells in the treatment of numerous diseases; in this case, chitosan helped to obtain a more stable complex [56]. In addition, several reports confirmed that chitosan nanoparticles loaded with onion extract [57], resveratrol, and ferulic acid [58] successfully induced apoptosis and possessed cytotoxicity activity against breast and skin cancer, respectively. 

As described, chitosan is a powerful material that helps protect nutraceuticals from premature degradation derived from different factors (e.g., light, pH, and temperature) and improves the bioavailability of these active compounds. Additionally, some formulations involving chitosan-based materials and nutraceuticals can be easily prepared.

### 3.2. Chitosan in Synthetic Drug Encapsulation: Anticancer Drug Formulations

**Figure 4:**
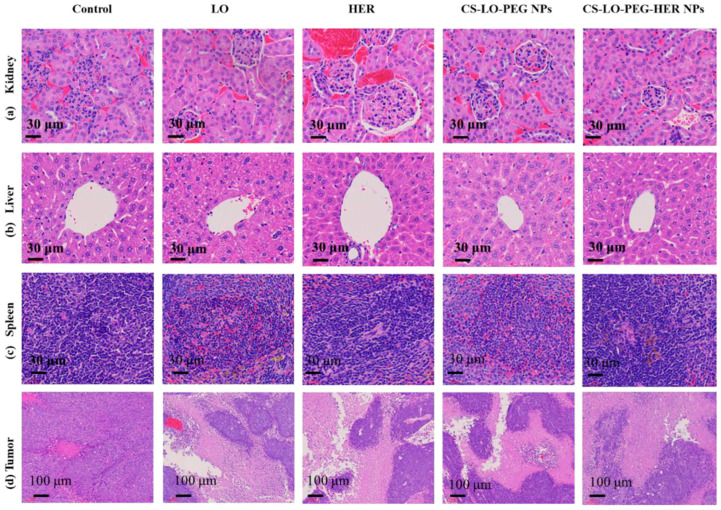
Effect of L-lysine α-oxidase (LO), herceptin (HER), and different chitosan (CS)-polyethylene glycol (PEG) nanoparticle treatments on histopathological changes in organs and tumor pathology: kidney (**a**), liver (**b**), spleen (**c**), and tumor (**d**). Image taken from the work by Saravanakumar et al. [72].

Cancer is a leading cause of death worldwide, and even though chemotherapy is one of the most effective methods for treating cancer, its clinical application needs to be improved due to the antineoplastics’ cytotoxicity, and the fact that many chemo-drugs are poorly water-soluble, lack of targeted delivery, have side effects, and experience drug resistance [59]. A drug delivery system intended for cancer should be biocompatible and maintain the drug’s therapeutic activity, delivering the antineoplastic to the target tissue in a controlled way. That could allow achieving the desired concentration while reducing systemic side effects and the therapeutic dose [21,60]. Among drug delivery systems, nanosized carriers are an outstanding approach because they have shown high drug loading efficiency and could penetrate tissues accumulating around the tumor [21]. For that, chitosan has been widely studied developing pH-responsive drug delivery systems, which swell in an acidic medium due to the protonation of amino groups onto the polysaccharide. Thus, this biocompatible polymer is suitable for drug delivery in cancer, where the tumor microenvironment exhibits a low pH due to the anaerobic metabolism [60]. In contrast to healthy tissues, during their progression state, tumors may exhibit a higher or lower temperature as a result of increased vascularization or reduced metabolic activity, respectively [61]. Hence, thermosensitive nanocarriers containing chitosan have been designed for cancer therapy. For that, chitosan grafted with poly(*N*-vinylcaprolactam) or poly(*N*-isopropylacrylamide) are the most-used copolymers in trials where the temperature is a triggering factor for drug release [62,63]; this responsiveness is derived from a reversible phase transition based on their lower critical solution temperature (LCST). Additionally, dual- and triple-stimuli responsive materials are created for achieving a targeted and controlled drug release. Examples of dual-responsive chitosan-based materials include those that combine temperature and pH-sensitive systems [64,65], as well as those that use pH and electric field-sensitive polymers [66] for triggering drug release. It has been proposed that chitosan can bind to small molecules like doxorubicin, where the binding free energy can depend on the degree of protonation when electrostatic repulsion with positively charged (DOX) takes place, so chitosan in a more deprotonated state allows stronger hydrogen bonds [19]. In the case of non-ionized molecules like 5-fluorouracil, hydrogen bonds have been proposed as the principal reason for the strong interactions between the drug and the chitosan-based carrier [20]. In other contributions, nanoparticles from modified chitosan encapsulating chemotherapeutics such as doxorubicin (encapsulation efficiency up to 85%), 5-Fluorouracil (encapsulation efficiency up to 86%), oxaliplatin, methotrexate, and paclitaxel (encapsulation efficiency up to 79%) have been developed and evaluated as a potential treatment for cancer [67,68,69]. Furthermore, chitosan-based micelles (particle size = 211 nm; drug loading capacity = 54%) have presented outstanding pH-triggered doxorubicin release with negligible premature drug leakage in 60 h, providing better tumor cell growth inhibition than the free drug [70]. Furthermore, micelles (average size < 200 nm; zeta potential = 43 mV) obtained with amphiphilic chitosan grafted with O-methyl-O′-succinyl polyethylene glycol (mPEG) and oleic acid were developed for oral administration of camptothecin (CPT; drug loading around 8%), helping to decrease colorectal cancer (CRC) progression. This platform improved the aqueous solubility of CPT and protected it from gastrointestinal conditions, resulting in anticancer activity against CRC cell lines (such as Caco-2 and HT29), and a significant decrease in tumor growth and inflammation was observed [71]. As an example of triple-stimuli responsive materials, a cascade-responsive nano-platform (particle size < 200 nm) was developed for breast cancer therapy. The system combined the thermosensitive characteristic of poly(*N*-vinylcaprolactam), the acidic pH response of chitosan, and the cell-penetrating peptide, attaining selective nanoparticle penetration in tumor cells for doxorubicin release; as a result of in vitro and in vivo trials, the formulation that had doxorubicin was selectively taken up by cancerous cells [67]. Figure 4 shows the results of in vivo tests using chitosan in formulations (PEG-HER NPs) against cancer; CS-LO-PEG-HER NPs were prepared from chitosan (CS), L-lysine α-oxidase (LO), polyethylene glycol 600 (PEG), and herceptin (HER). The authors stated that the nanoparticles presented cytotoxicity in BT474-xenograft tumor mice by promoting reactive oxygen species, mitochondrial membrane potential loss, and nucleus damage, resulting in a significant tumor cell reduction and avoiding damage in the kidney, liver, and spleen. Further, they indicated that the system enhanced the utilization of LO to achieve a promising anticancer effect [72]. As is known, PEG helps increase the penetration ability of the drug delivery system [73]. Additionally, chitosan functionalization has been exploited for tumor targeting focused on overexpressed surface molecules or receptors on the cancer cell membrane, and single receptor targeting has been developed [74,75]; however, a dual receptor is preferred to enhance penetration. For example, folate receptors (FR) and epidermal growth factor receptors (EGFR) are key markers for tumor tissues. An FR ligand is folic acid (essential for cell growth and DNA replication), and an EGFR ligand is cetuximab. For lung carcinoma treatment, docetaxel-loaded chitosan nanoparticles, decorated with the dual receptor targeting of FR and EGFR, showed improved bioavailability and half-life, achieving longer circulation time and sustained release [21]. In summary, chitosan-based materials have been tested and resulted in outstanding candidates for drug delivery systems toward controlled-targeted drug release, improving the bioavailability and reducing systemic side effects of antineoplastics. However, these formulations continue on the winding road of evaluations required before they can be considered for chemotherapy in patients.

## 4. The Role of Chitosan in Food: Material to Extend the Shelf Life

### 4.1. Coatings from Chitosan for Fruits and Vegetables

At the present time, besides the consumer interest in food quality and safe foods with new functionalities, it is required new materials with antimicrobial properties protecting fruits and vegetables during storage, which has extended the research concerning coating materials that can be fully eaten [76,77]. In addition, consumers demand foods with environmentally friendly packaging, forcing the industry to innovate and develop new packaging strategies. Chitosan fits perfectly with this challenge.

Edible coatings are thin layers made from edible materials that are formed into solid sheets and then applied over the food product [78]; these coatings help prevent moisture loss and microbial development, establishing a semi-permeable safety barrier and maintaining the product’s structural integrity. Eventually, they could contain antioxidants and antimicrobials as to avoid deterioration in food products [79]. Chitosan is considered a suitable material for the purposes of coating formation for fruits and vegetable protection because it is biodegradable and biocompatible, has biocidal activity and gas barrier properties, and yields edible coatings with excellent adhesiveness and cohesion with smooth surfaces for food products [80]. Authors have stated that suitable chitosan-based films do not alter the appearance, flavor, aroma, or texture of fruits (e.g., strawberries) [81]; in addition, this material helps control the oxidative stress, preserving a proper balance of reactive oxygen species in fruit cells [82]. Furthermore, chitosan allows an easy combination with additional components toward film formation, such as other polysaccharides, plasticizers, proteins, or lipids. Hence, this outstanding polymer favors the formation of coating and films with good mechanical properties that have selective permeability to oxygen [83]. Parameters such as tensile strength and elongation at break can be adjusted by using an optimal content of chitosan [84]. Researchers have indicated that a higher chitosan content (more free amino groups of the polymer) in composite films led to a more compact structure, decreased permeability, and enhanced antioxidant activity [85]. There are several methods to obtain chitosan edible coatings. The most popular is the casting, where the coating formation occurs owing to the preservation of chain entanglements and intermolecular interactions, such as electrostatic and hydrogen bonds, promoted during the drying process [86]. With this strategy, the tensile strength, swelling power, and greenness value can be controlled through chitosan concentrations and drying temperatures [87]. 

**Figure 5:**
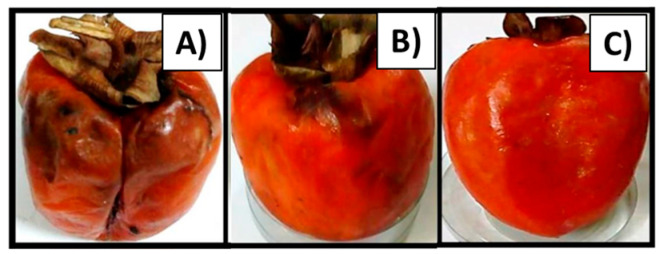
Illustrative example of edible coating: protection of persimmon fruits (*Diospyros kaki* L.) using edible coatings from nanochitosan (**B**) and rosmarinic acid-mediated selenium nanoparticles (**C**) compared with the uncoated fruit (**A**) after 14 days of storage; images were taken and combined from the literature [91].

As illustrative examples, from chitosan-based films plasticized with spermidine and/or glycerol, authors reported that the incorporation of spermidine increased markedly the elongation at break, just as proper concentrations of both spermidine and glycerol enhanced the extensibility and plasticity; also, the gas permeability (GP) was reduced (2.40 cm^3^ mm m^−2^ day^−1^ kPa^−1^) but the water vapor permeability (WVP) was higher (0.37 cm^3^ mm m^−2^ day^−1^ kPa^−1^), as compared with single chitosan (GP = 15.81 cm^3^ mm m^−2^ day^−1^ kPa^−1^; WVP = 0.05 cm^3^ mm m^−2^ day^−1^ kPa^−1^) [88]. Similarly, researchers carried out the preparation of alginate/chitosan-mixed edible films as a coating on figs (*Ficus carica*), and found that the coating preserves bioactive compounds and the antioxidant capacity of the product during storage [89]. In other work, Pavinatto et al. studied chitosan-based coatings for the mechanical and biological protection of strawberries, where glycerol was used to enhance elasticity and hydrophobic character. The results showed that fungal growth in coated strawberries was not detected (after 7 days at 23 °C) when chitosan/glycerol-30% films were used, but uncoated strawberries were completely taken up by fungi [81]. In the same sense, an edible antimicrobial coating was produced from chitosan modified with monomethyl fumaric acid (CS-MFA) for fresh strawberries; when compared with the non-modified polymer and the control samples, CS-MFA decreased the weight loss, total aerobic count, and the count of yeast and molds [90]. In another contribution, the protection of persimmon fruits (*Diospyros kaki* L.) was studied using edible coatings from nanochitosan (Figure 5B) and rosmarinic acid-mediated selenium nanoparticles (Figure 5C), obtaining better results with the nanocomposite containing rosmarinic acid/Se to prevent black rot disease and preserve firmness of fruits after 14 days of storage, compared with single chitosan and the uncoated fruit (Figure 5A). This result can be attributed to the synergistic effect of chitosan–rosmarinic acid-Se [91]. Thus, chitosan based-coatings are highly promising materials for extending the shelf life of fruits during storage.

Although the laboratory results are convincing, the application of chitosan in the packaging sector has not yet reached the stage of industrialization because its price is still high. However, this sector will develop in the coming years, for example, with the aid of the nanotechnology, which is an interesting approach in the formulation of active ingredients for packaging applications.

### 4.2. Biodegradable Plastics Containing Chitosan: Food Packaging

**Figure 6:**
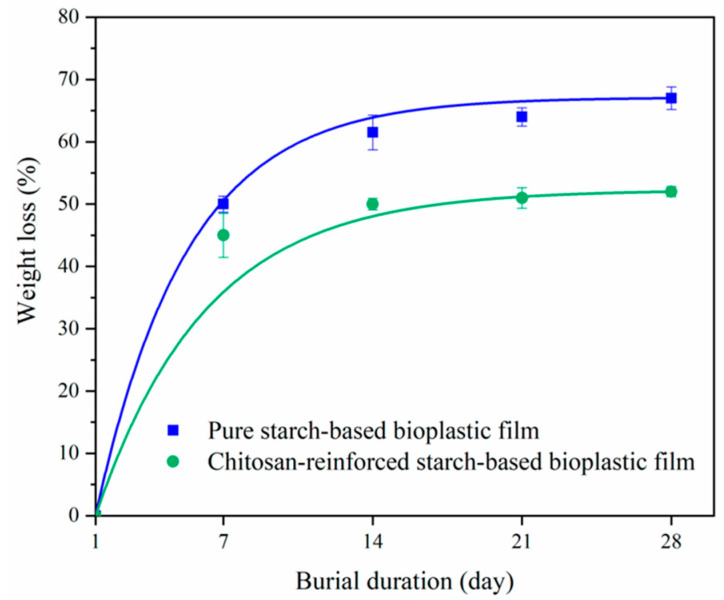
Weight loss percentages of biodegradable plastic films, based on starch and starch-chitosan, during a burial period. The figure was taken from the literature [18].

Biodegradable plastics are materials that can be broken down into water and CO_2_ by naturally occurring activities of bacteria, fungi, and algae. Thus, the degradation rate depends on the environments where they end up (e.g., soil or marine water) [92]. In this topic, several variables affect the biodegradability, including the raw material, chemical composition, final product structure, and the environmental conditions in which the product is expected to biodegrade [93]. Poly(lactic acid) (PLA), obtained from renewable sources, seems to be one of the most promising biodegradable materials for replacing plastics derived from petroleum, because this polymer provides similar or better properties than conventional plastics [94,95]. However, despite the biological compatibility and high transparency of PLA, properties such as high flammability, poor ultraviolet resistance, and brittleness need to be addressed [96,97]. To this end, biodegradable-renewable polysaccharide nanoparticles like cellulose, starch, chitin, and chitosan are a preferred alternative to use together with PLA, thus creating nanocomposites. Particularly, chitosan possesses additional advantages such as antimicrobial activity, the possibility of chemical modification from its reactive amino groups, and excellent functional properties when combined with other materials [98,99,100]. Adding different content (0–5.0% *w*/*w*) of chitosan nanoparticles to PLA by twin-screw extrusion benefits the properties of the resulting composite by enhancing the elongation and the impact strength; however, the tensile strength and thermal stability are decreased [101]. Another important aspect of these plastics is biodegradability; in this regard, a film based on PLA and chitosan was fabricated by a non-solvent induced phase separation method. The synthesized film presented a porous structure where the pore size can be changed by modifying the PLA/chitosan ratio, and more importantly, the degradation rates were proportional to the pore size; therefore, tunable degradation rate can be obtained [102]. The degradation behavior under different times of standard weathering conditions has been analyzed for a film containing polyethylene, PLA, and chitosan prepared by extrusion. The films containing a mixture of synthetic and natural polymers are more susceptible to degradation in comparison to films without chitosan. Moreover, the incorporation of a compatibilizer (poly (ethylene-g-maleic anhydride)) into the films increases the degree of homogeneity and favors film degradation without a significant effect on their thermal stability [103]. Chitosan microspheres and phytic acid with core-shell structure have been developed and employed as additives for PLA composites, which improved flame retardancy, mechanical properties, UV resistance, and degradation capacity in soil. To explain the accelerated degradation, the authors proposed that the water is easy to gravitate and attack the chain of PLA, and both the additive and water assisted the microbial reproduction, and these processes simultaneously erode the film [96]. In other work, Chang et al. prepared chitosan/PLA plastic films by extrusion and demonstrated that covering fish fillet with a 0.5% chitosan–PLA film reduced the number of several microbes (e.g., mesophiles, psychrophiles, coliforms, *Pseudomonas*, *Aeromonas*, and *Vibrio*) and the total volatile basic nitrogen value in the grouper fillets, when stored at 4 °C [104]. Similarly, composite films from nanochitosan in PLA matrix, using polyethylene glycol as a cross-linking agent and polyvinyl alcohol as a plasticizer, were found to be useful for the packaging of fresh prawn as it extended its shelf life. In this case, the quality parameters of the product were acceptable until 15 days of storage wherein the use of chitosan (1%) effectively reduced the microbial growth. Furthermore, the author indicated that both the film thickness and the chitosan incorporation influenced the permeability of the film [105]. Tan et al. developed biodegradable plastics from chitosan-reinforced starch-based films; they investigated the effects of processing parameters, such as the polymer concentration, glycerol loading, and temperature, on mechanical properties. As a result, a tensile strength of 5.19 MPa and elongation at break of 44.6% were attained using reaction conditions involving 5 wt.% starch, 40 wt.% glycerol, and 20 wt.% chitosan at 70 °C. Chitosan-reinforced films had a lower water uptake capability, as compared with pure starch-based films; furthermore, pure starch-based and chitosan-reinforced films are subjected to applications below 316 °C and 290 °C, respectively. Additionally, a biodegradation test was conducted in compost soil, obtaining that the bioplastic containing chitosan underwent slower degradation which might be due to the starch-chitosan interaction and the reduced hydrophilicity (Figure 6) [18]. Researchers have also demonstrated that chitosan can be uniformly integrated into polyethylene terephthalate (PET), a typical packaging material for disposable soft drink bottles, through the extrusion process. The best performance in miscibility and degradation was reached with a mixture 95/5 (PET/chitosan) in weight ratio [106]. 

The incorporation of chitosan into plastic materials modifies characteristics like UV resistance, transparency, and antimicrobial activity, among others. Moreover, the resulting plastic is more easily degraded; nevertheless, these biodegradable materials are often not as biodegradable as required. It is unlikely to find a unique solution in terms of designing a single polymer, which degrades easily in a wide variety of ecosystems.

## 5. Agriculture: The Role of Chitosan in Plant Growth

One of the goals of sustainable development is to ensure food for all people worldwide. Thus, it is necessary to improve food and agriculture systems. In this regard, chitosan has been registered with EPA (US Environmental Protection Agency) as a fungicidal and antimicrobial agent, as well as a plant growth regulator (PGR) within the minimum risk pesticide list [107]. The advantage of chitosan appreciated by farmers is its contribution to promoting plant growth, eliciting plant resistance against biotic and abiotic stress, and activating symbiotic signaling between plants and beneficial microorganisms [108]. Chitosan and its fragments have been shown to act as defense elicitors for diseases, mainly fungal infections, as they are recognized by the plant as stress signals. For instance, plants increase hormones and phenolics production when chitosan is applied to plants’ roots [109]. Foliar spraying of chitosan solutions also impacts the infection by fungal pathogens; as an example, it has been reported that *Botrytis cinerea* infection is affected by the increase of plant resistance when chitosan is applied. This effect is related to callose deposition and accumulation of jasmonic acid (JA) in leaf tissues [110]. Chitosan has also been studied as a growth promotor. Studies on ornamental plants like *Dendrobium* orchids indicated that this polysaccharide increases floral production by affecting chloroplast gene expression [111]. For that, its oligosaccharides of low molecular weight chains are recognized to be more active [112]. When applied to baby leaf red perilla (a culinary vegetable), chitosan promoted plant height, fresh weight, and antioxidant levels, acting as a biostimulant for plant growth and quality [113]. For the in vitro germination of plants, the supplementation of media with plant growth regulators is used to stimulate seed germination and organ development. Studies have shown that chitosan and its oligomers can be used as alternatives to the commonly used plant growth regulators including auxins and cytokinins [15]. In this regard, germination studies conducted on orchids confirmed that chitosan acts as an in vitro growth stimulator for meristemic tissues, accelerating protocorm formation up to 15 times compared to control plants, and demonstrating the relationship between the polymer molecular weight and its effectiveness [112]. Chitosan nanoparticles obtained with TPP have also been studied as elicitors for germination but these have shown a phytotoxic effect at lower concentrations (5–20 mg L^−1^) than the bulk chitosan (100 mg L^−1^), causing a dramatic growth cessation. Bulk chitosan achieved higher antioxidant levels; nevertheless, the nano-chitosan was the most effective elicitor for organogenesis [114]. Similarly, the results of chitosan microparticles supplementation improved the promotion of the foliar area and root and shoot biomass than bulk chitosan in tomato seeds [115]. Chitosan oligomers can enhance the activity of enzymes involved in primary (e.g., nitrate reductase, ribulose-1,5-bisphosphate carboxylase/oxygenase, and carbonic anhydrase – all of these are involved in photosynthesis) and secondary (e.g., phenylalanine ammonia lyase and L-tryptophan decarboxylase, for phenolics and alkaloid biosynthesis, respectively) metabolic pathways [116,117]. The secondary metabolites “terpenes” are economically attractive for their curative and industrial uses, and their production in plants also increases with the application of chitosan [116]. This metabolic response to chitosan has been related to the miRNA and mRNA expression in plants [118]. In the same way, the authors studied the foliar application of a mixture of semisynthetic chitosan derivatives to induce tolerance to water deficit (for 15 days) in maize, finding that the mixture of derivatives increased the content of phenolic compounds and the activity of enzymes involved in their production, increasing dehydroascorbate reductase, total soluble sugars, total amino acids, starch, grain yield, and harvest index [119]. 

**Figure 7:**
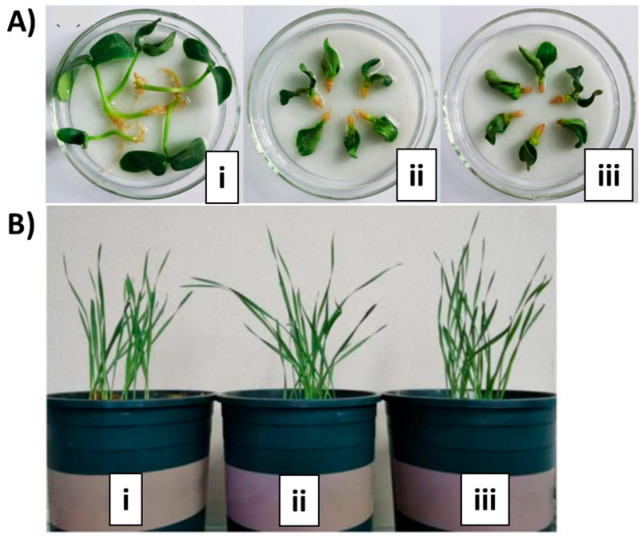
Bioactivity for the target cucumber plant (**A**) and non-target wheat plant (**B**), involving a study with carboxymethyl chitosan for controlled release of pesticides; control with deionized water (i), free pesticide (ii), and pesticide-loaded micelles (iii). Adapted from a previously reported work [23].

In this topic, materials from chitosan can also be tailored for encapsulation and slow release of plant growth regulators (e.g., pesticides and fertilizers), being a polymeric matrix that provides different benefits, such as protection of guest compounds from adverse environmental conditions (pH, light, temperatures) and protection of plant cells from hazardous effects thus avoiding a burst release of active ingredients [120]. For instance, Feng et al. studied coumarin-containing light-responsive carboxymethyl chitosan nanocarriers for controlled release of pesticides (see Figure 7), and found good bioactivity on the target plant (cucumber) with no impact on the non-target plant (wheat) using 2,4-dichlorophenoxyacetic acid as model pesticide [23]. Based on the abovementioned, chitosan application in crops, medicinal, and ornamental plants influences plant defense and plant growth by inducing enzymatic genes for primary and secondary metabolism, and it is a promising way for increasing the yield of economically valuable secondary products.

An interesting recent approach is genetic engineering using nanochitosan for a sustainable increase in crop productivity. Results showed that nanochitosan enhanced anti-pathogenic and plant growth-promoting activity. Nanochitosans are also promising materials in agriculture for the controlled release of pesticides, nutrients, fertilizers, and plant hormones [121,122].

## 6. Textile Industry: Development of (Cosmeto-)Textiles Containing Chitosan

**Figure 8:**
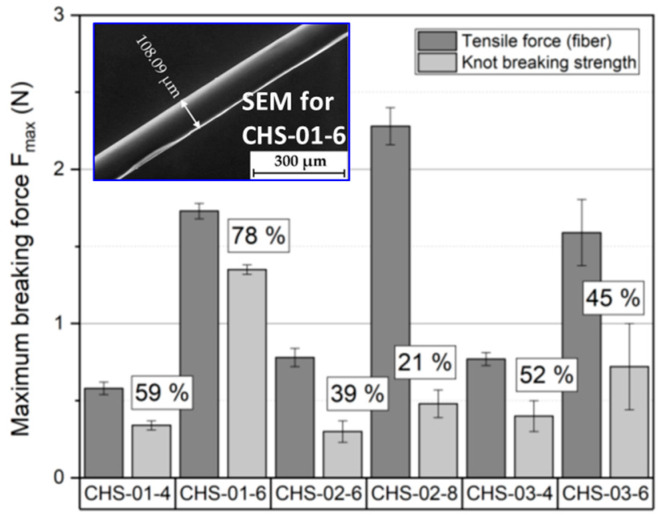
Textile physical tests of chitosan-based fibers, comparing the maximum tensile force and maximum knot breaking strength after a knot formation for the fibers from chitosan and ionic liquid 1-butyl-3-methylimidazolium acetate; the figure was taken and combined from a previously reported work [127].

The demand for textile goods with antimicrobial activity is continuously growing, and a number of chemicals are used to fulfill this task; nevertheless, the change from toxic to non-toxic chemicals, producing eco-friendly materials, is preferred. For instance, medical staff, law enforcement officers, and firefighters, among other occupations, should be correctly protected from biological agents to avoid the propagation of infectious microorganisms [123]. In this sense, chitosan is a versatile polymer with broad applications in the textile industry [1]. The main characteristics of chitosan appreciated by textile industrialists are its biodegradability, antistatic activity, chelating property, deodorizing property, ability to form films, chemical reactivity and encapsulating capability, ability to control the strength and rigidity of fibers and dyeing, thickening properties, and its ability to heal wounds, the latter being of interest in the biomedical field (surgical threads and sanitary fibrous products) [124,125]. Specifically, chitosan can be used to concede excellent properties like antimicrobial activity to commercial textiles; this power against several bacteria and fungi is due to its polycationic nature [126]. Authors have performed textile physical tests of chitosan-based fibers, comparing the maximum tensile force and maximum knot breaking strength after a knot formation for the fibers from chitosan and ionic liquid 1-butyl-3-methylimidazolium acetate (see Figure 8), where both parameters were adjusted by controlling the chitosan content [127]. Furthermore, cotton and wool textiles pretreated with chitosan present good affinity to anionic dyes, high dye uptake, and color strength due to the high proportion of amino group on chitosan, which provided more adsorption sites for anionic dyes through van der Waals forces and electrostatic attraction [128]. This property of chitosan is highly relevant in two different aspects; first, the amount of dye required in the process is less, and therefore, it is more economically viable; second, the dye deposited into the environment is reduced. When dyes are released into the environment, they can generate lethal wastes, and also, they are extremely mutagenic and carcinogenic. Besides, their perseverance endangers productive agricultural land and aquatic life, and even a small dye concentration adversely affects gas solubility and the transparency of water [22]. Chitosan is incorporated in textile products as fiber after undergoing a series of processes. However, the fibers have some limitations related to poor mechanical properties, high electrostatic charge, and high cost [129]. To overcome these limitations and enhance the performance of the manufactured yarns, distinct approaches have been addressed during its synthesis, such as the blending ratio of chitosan with a second material, blending methods, the solvent used, and others [130]. During textile production, a sizing agent is needed to protect against breaking the fibers and filament yarns in the weaving machine; for that, chitosan has been proposed as a highly compatible alternative to synthetic sizing agents. The economic and ecological advantages of applying chitosan in sizing were demonstrated with the weaving efficiency increase (based on the reduction in yarn breakage), reduction in wastewater (from the use of less sizing agent), and eco-friendly textile production (from substances which are easily biodegradable) [131].

In this topic, hydrogels from sodium alginate and chitosan formed on textile nonwovens (textile-hydrogel hybrids) were designed for use in mild-to-moderate exudate wounds (e.g., ulcers and burns): for that, formulations with the lower hardness, compressibility, and adhesiveness were selected to be applied to textile nonwovens [132]. Grgac et al. reported that chitosan particles are well implemented in the cotton and polyester/cotton blend fabrics, where the antimicrobial activity after five washing cycles was persistent [133]. The shrinkage-proof property of wool substrate modified with chitosan-poly(propylene imine) dendrimer hybrid has also been addressed, where the shrinkage of the untreated fabrics was larger in both directions (warp and weft), as compared to chitosan-poly(propylene imine) treated fabrics. This behavior was attributed to the fact that fibers containing chitosan would become stronger to external forces and not slip over each other, thus becoming more shrink-proof [134]. In other work, a water-soluble chitosan derivative, *O*-acrylamidomethyl-*N*-[(2-hydroxy-3-dimethyldodecylammonium) propyl], was synthesized and applied to cotton samples. The treated cotton fabrics were able to maintain antimicrobial properties against *Escherichia coli* even after 30 home launderings. Furthermore, salt-free reactive dyeing of the treated fabric showed good dyeing properties and washing fastness [135]. Researchers have also tested chitosan microcapsules containing an antifungal agent (clotrimazole) with potential applications onto socks or bandages as a treatment for athlete’s foot; the performance of these microcapsules was evident after studying the in vitro inhibition of *Trichophyton rubrum* growth and cytotoxicity (in skin cell lines). The authors suggested that the system could continuously release antifungal agents in a controlled manner under pressure [136]. Other chitosan microcapsules loaded with miconazole nitrate (encapsulation efficiency = 77.6–96.8%) have been successfully prepared, releasing around 50% of the drug after 12 h under conditions that mimic human skin. The authors proposed that the formulation could be applied onto bandages or socks [137].

As demonstrated by the studies mentioned earlier, Chitosan is a promising polymer in the development of (cosmeto-)textiles due to its versatility. Nonetheless, there are still some challenges that need to be tackled with the aim of considering chitosan for textile production at an industrial scale. In addition, for cosmetotextiles, it is important to conduct scientific studies in order to promote the use of chitosan on the basis of proven results and not only attributed to fashionable effects. A commercial example is anti-cellulite slimming leggings, which allow slimming without or almost without effort.

## 7. Synthesis Processes: Chitosan in Catalytic Scaffolds

Catalysis is a technology developed to increase the rate of chemical reactions and/or establish mild reaction conditions using catalysts. Ninety-five percent of chemicals in the industry come from catalytic processes [138]. In this field, heterogeneous catalysis offers advantages over its counterpart (homogeneous), such as easier separation of catalyst from the reaction mixture, reusability, good stability, and low toxicity of catalyst, among other factors [139]. Regarding solid catalysts, the supported ones are the most commonly used, and they are typically formed by nanometric particles of at least a metal that is dispersed on the support’s surface [140,141]. In this topic, chitosan is an attractive material to be used as support in supported catalysts. The presence of amino and hydroxyl groups onto the polymer chain provides a number of structural modifications that can improve thermal and mechanical properties [142]. Besides, chitosan functional groups represent possible binding sites that could strongly interact with metal ions and metal nanoparticles [31,143], which is a key requirement for a catalyst support. Recently, the excellent properties of TiO_2_ as a photocatalyst for the degradation of different organic compounds in an aqueous medium have been reported, but one of the challenges for its technical implementation is the difficulty in its separation from water. Therefore, to address this challenge, Bergamonti et al. employed a 3D-printed chitosan scaffold as a support for TiO_2_ and obtained an active photocatalyst for amoxicillin degradation. The size of the TiO_2_ nanocrystals (approximately 20 nm for the anatase phase and 25 nm for the rutile phase) was not affected by its immobilization within the 3D chitosan scaffold [144]. Furthermore, chitosan-based scaffolds supporting metal nanoparticles have been claimed to be active for catalytic reduction in pollutants; for that, Pt and Pd nanoparticles were in situ formed into walls of 3D-macroporous scaffolds (cryogels), resulting in materials catalytically active to 4-nitrophenol reduction [31]. Thus, 3D-chitosan scaffolds seem to be suitable versatile supports for catalytic/photocatalytic applications. Another interesting catalytic topic involves the conversion of biomass to high-value chemicals or fuels, where lignin, cellulose, and hemicelluloses are the three major components of lignocellulosic biomass. Recently, photocatalysis has emerged as a promising method for that purpose [145], and it has been reported that chitosan-based catalysts are efficient photocatalysts. Li et al. reported a feasible path for lactic acid production via photocatalytic reformation of biomass, promoted by an alkaline chitosan hydrogel hybridized with CuO [146]; these authors attributed the efficiency to the CuO ability for visible-light adsorption (CuO band gap is ca. 1.7), and to the improved stability of CuO provided by chitosan; additionally, the visible-light adsorption of CuO was not affected after hybridization with chitosan. Other catalytic processes also require improved thermal stability and mechanical properties of chitosan to be used as support. That can be performed by cross-linking and bonding chitosan with other macromolecular chains [142]. For instance, Rostami et al. designed and synthesized a thermally stable network by cross-linking chitosan and cellulose using EDTA, Cs-EDTA-Cell [147]. The authors tested the chitosan-based network as a catalyst for the synthesis of 2-amino-4*H*-pyran derivatives at room temperature; they found that catalysts promoted high yields of the desired products in short reaction times. 2-amino-4*H*-pyran is one of the most biologically active scaffolds in medicinal chemistry, with potential pharmaceutical applications as anticancer, anti-HIV, anti-inflammatory, etc. On the other hand, the hydrogenation of CO_2_ to hydrocarbons has been widely investigated [148,149]. As catalytic CO_2_ hydrogenation demands moderate to severe reaction conditions, pure chitosan cannot be used as a support. However, the ability of chitosan to stabilize metal ions and metal nanoparticles is strongly beneficial to the green synthesis of catalytic materials for that purpose. Witoon et al. reported the important role of chitosan in the physicochemical properties and catalytic activity of Cu/ZnO for CO_2_ hydrogenation to methanol (a fuel) [150]. The authors employed chitosan as a precipitating agent during the co-precipitation of CuO and ZnO, finding that chitosan acted as a soft template for the formation of hollow nanospheres. 

Chitosan also plays a very important role in catalysis as a green solvent or green electrolyte. It generates green solvents, increasing the surface exchange capabilities and utilization of ionic liquids, contributing to the implementation of green chemistry principles by minimizing the amount of required products and the utilization of renewable raw materials. These solvents open up new fields of non-aqueous biocatalysis and biotechnology (enzymology).

To conclude, it is clear that some of the main challenges to society involve global food supply, global warming, environmental pollution, production, and use of clean and renewable fuel and chemical platforms. Catalysis is deeply related to all these topics. Based on its physicochemical properties, abundance, and low toxicity and cost, chitosan represents a potentially sustainable and versatile material for catalytic applications.

## 8. Water Treatment Using Chitosan: Flocculation

**Figure 9:**
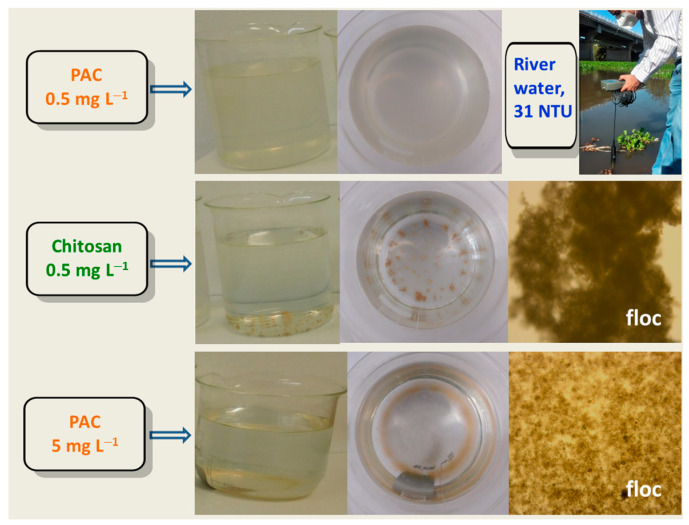
Turbidity reduction in water samples from the Culiacán River using chitosan and commercial poly(aluminum chloride) (PAC). Images were captured using a digital camera and a light microscope (magnification 10×) after flocculation trials. The images were taken in the Environmental Laboratory of the Faculty of Engineering Culiacán-UAS, Mexico, by Diana V. Félix-Alcalá.

Water treatment/remediation is an issue of great interest to ensure the water supply for future generations. In recent years, the increase in contamination of natural water reservoirs (e.g., rivers and lakes) has been exposed [151]. As is well known, water treatment plants are inefficient in removing certain substances such as metals, drugs, dyes, plastics, and pesticides because they were not designed to remove these pollutants [152,153]. Furthermore, the inappropriate management of effluents triggers freshwater contamination resulting in an ecological disturbance and representing a public health risk [154,155]. Therefore, it is essential to improve the processes involved in the treatment trains. Chitosan, a low-cost and versatile biopolymer, can be used for environmental applications, including water and wastewater treatment (biocoagulation, bioflocculation, and biosorption), membrane filtration (polymer-assisted ultrafiltration), sludge dewatering, and odor reduction. Among them, flocculation deserves particular attention; for example, for the removal of pollutants present in water from aquaculture [156]. The flocculation process is widely used at the industrial scale, where eco-friendly flocculants such as chitosan with high effectiveness, manufactured from renewable sources, and ease of use are desired [157]. It is relevant to highlight that unmodified chitosan has presented a higher performance potential than poly(aluminum chloride) (PAC) when pH regulation and the removal of heavy metal ions from wastewater were studied, which could be attributed to the great effectiveness of this aminopolysaccharide in the removal of dissolved/dispersed organic matter (combining coagulation and flocculation), in addition to its high chelating ability [158]. Moreover, turbidity reduction using samples from rivers and wastewater has been quite comparative for both flocculants, obtaining bigger and more compact floc with chitosan (see Figure 9); further, in the case of metal ion removal, chitosan has shown more affinity to certain metals. However, an optimal dose (e.g., from 0.5 to 15 mg L^−1^) is usually required [16,158]. The simultaneous addition of PAC and chitosan to low turbid water resulted in efficient turbidity removal of 87%, indicating a synergistic effect between the two polymers [159]. The use of modified chitosan, such as chitosan-based graft copolymers, can result in water-soluble materials that exhibit a wider flocculation window, a range of concentrations yielding high effectiveness, and higher flocculation performance toward the removal of turbidity, small molecules, and heavy metal ions [160]. For instance, chitosan-based graft copolymers have also been proposed as good alternatives to replace commercial adsorbents for the removal of textile dyes [128]. With graft copolymers, the flocculation performance and floc characteristics are controlled using an appropriate number of chains grafted onto the chitosan backbone, that is, by adjusting the chemical composition [161]. Furthermore, the modified chitosan provides a synergistic effect with FeCl_3_ yielding higher turbidity and orthophosphate removal (>93%) with greater efficiency over unmodified chitosan [13]; for microplastic (polystyrene), the system tannic acid–chitosan conjugates and FeCl_3_ has shown higher removal efficiency (84%) aided by metal-polymer coordination bonds, as compared to single chitosan (54%) and tannic acid–chitosan (52%) in absence of Fe^3+^ [162]. On the other hand, the pH strongly affects the flocculation process when polymers susceptible to protonation and deprotonation are used. Therefore, the removal of turbidity and organic pollutants using chitosan-based flocculants is sensitive to the pH of the medium. From experiments using a river water matrix with different pH levels, the maximum flocculation efficiency using chitosan was reached at pH 7 [163]. Similarly, using chitosan-*graft*-(*N*-vinylcaprolactam-acrylic acid) for ciprofloxacin removal at pH 4, 7, and 9, a lower efficiency was registered at pH 4, because the polymer and drug were protonated, and this resulted in repulsive forces. At the same time, the higher entrapment was attained at pH 7, and that level was close to the isoelectric point of the flocculant and the lower drug solubility; therefore, the higher performance was related to additional interactions by hydrogen bonds among the polymer and drug molecules [164]. Thus, for water samples having pH values from 6.5 to 8, electrostatic interactions between chitosan (isoelectric point within the pH range 7–8) and contaminants are limited [158]. Regarding the temperature, it has been demonstrated that the antibiotic removal from wastewater can be improved by grafting thermoresponsive side chains onto chitosan, given that the hydrophilic/hydrophobic balance is changed when the temperature rises to values higher than the lower critical solution temperature of the flocculants [160,164]. Due to their nature, chitosan-based flocculants trigger a separation process that is mainly driven by charge neutralization (interaction with oppositely charged substances) and bridging (flocculant-contaminant adsorption forming complex aggregates) mechanisms [157]. In addition to other advantages in the flocculation process, these graft copolymers offer excellent sludge dewatering (improved sediment consolidation) [165]; also, when this type of flocculants have been regenerated, they have resulted in reusable materials which maintain high flocculation efficiency [160]. For their application on an industrial scale, the process using chitosan and its derivatives could be performed in the same infrastructure of treatment plants as for PAC, given that their dosage can also be carried out in a liquid formulation. Eventually, a chitosan-PAC mixture could be a feasible option in terms of its effectiveness, availability, and cost of materials.

Regarding the performance in other treatment methods, chitosan-based membranes have been designed for small substances entrapment via filtration, and in specific trials up to 81.21% of dye rejection (reactive black 5) and 78% of heavy metal removal (manganese) have been reached [166]. Hence, it is clear that this polysaccharide can help in the production of drinking water and water remediation by different strategies.

As a summary of the topics studied in this review, Table 1 shows additional examples of results obtained using chitosan-based materials for bacterial growth inhibition, drug and phytochemical encapsulation, edible coatings and food packaging, foliar application and pesticide encapsulation, fabrication of textiles, catalytic systems, and water treatment.

A recent comprehensive book on the applications of chitin and chitosan for environmental purposes has been published by Crini [183]. This book assesses their applications in water and wastewater treatment for sustainable solutions, and the future chitin and chitosan usage as an organic solution for a more sustainable, green, and healthy planet.

## 9. Conclusions

For the potential applications reviewed, the effectiveness of chitosan has been clearly demonstrated. Polymer molecular weight and degree of deacetylation, and in some cases its concentration, play a key role in influencing the effectiveness of this biocompatible polymer for different purposes. In some cases, the modification of the polysaccharide yields a remarkable increase in efficiency; these things considered, chitosan can be used either as the main component or as an adjuvant along with other materials. The hydroxyl and amino groups, as well as chemical modifications on the chitosan backbone, promote suitable binding with small molecules, ions, macromolecules, cell membranes/walls, and metal surfaces through electrostatic interactions, hydrogen bonding, hydrophobic interactions, and chelation. For some applications, there are no additional requirements for chitosan, but the challenge might be to ensure the global demand on an industrial scale where large volumes would be continuously required. In other cases, it is necessary to continue studying the components (substances) that achieve a better synergistic effect with chitosan, resulting in a better system. From a global perspective, chitosan could become a common chain connecting human health and environmental protection with a proper lifestyle.

## Figures and Tables

**Table 1:** Summary of representative results obtained with chitosan-based materials in different applications.

Material/System	Purpose	Result	Ref.
Chitosan-silver nanoparticles (Ch-AgNPs)	Bacterial growth inhibition in food	*Escherichia coli* was more susceptible to Ch-AgNPs than *Salmonella typhimurium*. *In vivo* antibacterial activity against *Escherichia coli* revealed excellent activity compared with single chitosan	[167]
Hollow nanoparticles from chitosan and alginate	Bacterial growth inhibition	Flexible capsules inhibited microbial growth more strongly than rigid particles. The inhibitory effect was from 18.6% to 34.9% for *Staphylococcus aureus* and from 23.7% to 40% for *Escherichia coli*	[168]
Conventional liposomes-triamcinolone acetonide coated with chitosan	Topical drug delivery system	High encapsulation efficiency (74%), suitable particle size of 176 nm, high positive surface charge (+41.1 mV, high stability), increased retention time, and maximum drug release of around 73%	[169]
Chitosan-gellan nanocapsules containing tamoxifen citrate	Drug encapsulation for breast cancer therapy	Spherical shape with particle size = 242 nm and zeta potential = 39 mV (value usually associated with high stability), providing sustained drug release and increased cytotoxicity against breast cancer cells (∼90%)	[170]
Chitosan nanoparticles containing *Physalis alkekengi-L* extract	Phytochemicals encapsulation: antioxidant compounds	Suitable particle size = 196 nm, zeta potential around 8 mV, and high percentage of encapsulated extract, close to 95%; resulting in improved stability and antioxidant activity of the *P. alkekengi-L* extract	[171]
Succinyl-chitosan nanoparticles	Phytochemicals encapsulation: antioxidant compounds	Encapsulation efficiency of 88%, 65%, and 27% for gallic acid, epigallocatechin-3-gallate, and propyl gallate, respectively. Encapsulation process governed by both the ability to form hydrogen bonds and the size of the guest molecules	[172]
Liposomal chitosan emulsions containing thyme essential oil	Phytochemicals encapsulation in edible coating	Emulsions were stable over 2 months at 4 °C. The Karish cheese preserved with the edible coating showed antimicrobial activity over 4 weeks, thus the shelf life of the product was extended	[173]
Chitosan-thyme essential oil films	Film containing in food packaging	Excellent antifungal activity against *Clonostachys rosea*. Conservation of fruit firmness, nutritional composition, and nutraceutical content, resulting in improved shelf life of Hass avocadoes	[174]
Corn starch–chitosan	Biodegradable film as packaging for food	Chitosan interacts effectively with starch, improving tensile strength, thermal stability, hydrophobicity, water adsorption capacity, and the gas barrier of starch films	[175]
Cross-linked chitosan/soybean protein isolate/polyvinyl alcohol	Hybrid plastic for packaging	Excellent compatibility of chitosan and soybean protein reducing the plastic surface roughness and enhancing mechanical properties, yielding superior water resistance compared to pure PVA. Hybrid plastic with desirable degradability	[176]
Chitosan	Foliar application	Reduced adverse effects of limited irrigation on essential oil yield, improved essential oil content, and positive influence on the amount of secondary metabolites. The antioxidant activity of sage (*Salvia officinalis* L.) was increased	[177]
Chitosan-tripolyphosphate nanoparticles containing nicotine hydrochloride	Insecticide encapsulation	Encapsulation efficiency of 55%, physicochemical stability (45 days) with particle size around 300 nm, and zeta potential close to 50 mV. Less than 20% of the insecticide was released within 24 h. The 24 h mortality of the formulation was 95% (against *Musca domestica*)	[178]
Chitosan-spinosad formulation	Insecticide encapsulation	High encapsulation efficiency (60%). Long sustained-release time (>18 days) and high cumulative release (>80%). Outstanding UV shielding ability of chitosan protecting spinosad from photodegradation	[3]
Emulsions from chitosan and alpha-tocopherol	Impregnation of cellulosic fabric for cosmetotextiles	Treated fabric with a slight decrease in absorbency and tensile strength, and good antibacterial (against *Escherichia coli* and *Staphylococcus aureus*) and antioxidant activities (36.78 unit g^−1^)	[179]
Nanocomposites based on chitosan/silver/clay	Treatment for cotton fabrics	Uniform morphology, high strength, flame retardant, high water absorption, high antimicrobial activity (against *Escherichia coli* and *Staphylococcus aureus*, >98%), controlled release of Lavender oil (odor retention even after 3 months), and UV protection	[180]
Scaffolds (imidazolium-vanillyl-chitosan Schiff bases (IVCSSBs)) for supporting Pd(II)	Catalytic systems	Heterogeneous catalyst with high catalytic activity (up to 99%) and stability in the reaction medium. Reusable materials with comparable catalytic activity after five operation runs. Excellent selectivity toward the Suzuki cross-coupling reaction	[181]
Cross-linked carboxyl-grafted chitosan derivatives	Wastewater treatment	Higher diclofenac removal (92.8%) using chitosan grafted with *trans*-aconitic acid, compared to succinic anhydride (80.9%) and maleic anhydride (66.2%) as grafting agents. Higher removal for diclofenac from a mixture with salicylic acid, ibuprofen, and ketoprofen	[182]

## Data Availability

Not applicable.
